# Common Genetic Variants near the Brittle Cornea Syndrome Locus *ZNF469* Influence the Blinding Disease Risk Factor Central Corneal Thickness

**DOI:** 10.1371/journal.pgen.1000947

**Published:** 2010-05-13

**Authors:** Yi Lu, David P. Dimasi, Pirro G. Hysi, Alex W. Hewitt, Kathryn P. Burdon, Tze'Yo Toh, Jonathan B. Ruddle, Yi Ju Li, Paul Mitchell, Paul R. Healey, Grant W. Montgomery, Narelle Hansell, Timothy D. Spector, Nicholas G. Martin, Terri L. Young, Christopher J. Hammond, Stuart Macgregor, Jamie E. Craig, David A. Mackey

**Affiliations:** 1 Genetics and Population Health, Queensland Institute of Medical Research, Brisbane, Australia; 2 Department of Ophthalmology, Flinders University, Adelaide, Australia; 3 Department of Twin Research and Genetic Epidemiology, King's College London School of Medicine, St Thomas' Hospital, London, United Kingdom; 4 Centre for Eye Research Australia, University of Melbourne, Royal Victorian Eye and Ear Hospital, Melbourne, Australia; 5 University of Tasmania, the Eye Hospital, Launceston, Australia; 6 Center for Human Genetics, Duke University Medical Center, Durham, North Carolina, United States of America; 7 Department of Biostatistics and Bioinformatics, Duke University Medical Center, Durham, North Carolina, United States of America; 8 Centre for Vision Research, Westmead Millennium Institute, University of Sydney, Sydney, Australia; 9 Lions Eye Institute, University of Western Australia, Centre for Ophthalmology and Visual Science, Perth, Australia; Georgia Institute of Technology, United States of America

## Abstract

Central corneal thickness (CCT), one of the most highly heritable human traits (h^2^ typically>0.9), is important for the diagnosis of glaucoma and a potential risk factor for glaucoma susceptibility. We conducted genome-wide association studies in five cohorts from Australia and the United Kingdom (total N = 5058). Three cohorts were based on individually genotyped twin collections, with the remaining two cohorts genotyped on pooled samples from singletons with extreme trait values. The pooled sample findings were validated by individual genotyping the pooled samples together with additional samples also within extreme quantiles. We describe methods for efficient combined analysis of the results from these different study designs. We have identified and replicated quantitative trait loci on chromosomes 13 and 16 for association with CCT. The locus on chromosome 13 (nearest gene *FOXO1*) had an overall meta-analysis p-value for all the individually genotyped samples of 4.6×10^−10^. The locus on chromosome 16 was associated with CCT with p = 8.95×10^−11^. The nearest gene to the associated chromosome 16 SNPs was *ZNF469*, a locus recently implicated in Brittle Cornea Syndrome (BCS), a very rare disorder characterized by abnormal thin corneas. Our findings suggest that in addition to rare variants in *ZNF469* underlying CCT variation in BCS patients, more common variants near this gene may contribute to CCT variation in the general population.

## Author Summary

Central corneal thickness (CCT) is an important eye measurement. It has been considered as a prognosticator for the development of glaucoma, with a thin cornea potentially increasing the risk of developing a subtype known as open-angle glaucoma. CCT is highly heritable, yet its genetic determinants are poorly characterized. We have revealed two loci near gene *FOXO1* and *ZNF469* associated with CCT in this multi-stage genome-wide association study examining over 5,000 samples. It is of particular interest that, while rare mutations in *ZNF469* cause Brittle Cornea Syndrome, more common variants near this gene also contribute to CCT variation in the general population. Furthermore, given the relation between CCT and glaucoma, results from our CCT studies will implement the search for the disease-susceptibility genes of glaucoma.

## Introduction

Glaucoma is one of the leading causes of irreversible blindness worldwide. It is estimated that by 2010, approximately 60.5 million people globally will be affected by this condition [1]. It is characterized by a progressive loss of retinal ganglion cells which will lead to visual field damage. The most common form is open-angle glaucoma (OAG), an adult-onset condition that generally affects people over the age of 40. Whilst several well-established risk factors including high intra-ocular pressure, ethnicity, increasing age and positive family history have been identified for OAG, recent evidence indicates that a decreased CCT is also a major risk factor. Numerous studies have shown that people with a thin cornea have a substantially increased risk of developing OAG and its associated visual loss [2]–[4]. It is conceivable from these data that there may be common genes in the pathways of both CCT and OAG [5]. Therefore, CCT is considered a useful quantitative trait for further dissecting the genetic aetiology of OAG. CCT is also an important measure in determining a person's suitability for laser refractive surgery to correct myopia and it may be abnormal in a range of corneal diseases such as corneal dystrophies, keratoconus and herpes simplex keratitis.

CCT is one of the most heritable human traits; the estimated heritability from existing studies is up to 0.95 [5]. Despite the strong evidence for a genetic background, the genetic architecture of corneal thickness remains unknown. Some candidate gene analyses have been conducted to assess the contribution of particular genes to this trait. For example, the candidates *COL1A1* and *COL1A2* encoding type I alpha collagens were determined based on the extreme CCT values observed in the connective tissue disorder osteogenesis imperfecta (OI) [6], [7] as explored by Dimasi et al [8]. Other possible candidates are the type V collagen genes *COL5A1*and *COL5A2* involved in Ehlers-Danlos syndrome (EDS) [9], fibrillin-1 gene (*FBN1*) responsible for Marfan syndrome [10], *PAX6* associated with aniridia [11] and *FOXC1* associated with abnormal ocular development [12]. Candidate gene analyses for normal CCT variation have been focused on the already known associations of disease genes with abnormal CCT; Genome–wide association (GWA) studies, on the other hand, can identify new genetic relationships without bias to known biology or disease associations of annotated well studied genes.

We carried out a multi-stage study on a panel of over 5,000 individuals in order to detect the genetic variation for CCT. In the first stage, we conducted GWA studies on the two twin cohorts from Australia and the UK separately. The first stage meta-analysis on the twin cohorts uncovered several promising chromosomal regions associated with CCT. We conducted another set of GWA studies on two population-based cohorts utilizing sample pooling techniques prior to genotyping (pool genotyping design) and developed a new analytical technique that allows comparison of the results from the different study designs. The findings were further validated by individually genotyping an extended cohort (pooled samples plus additional samples with extreme CCT values). Sample size of each study population and schematic of the study design can be found in Table S1 and Figure S1 respectively.

## Results

### GWA results from twin cohorts

CCT values in both twin cohorts are normally distributed with a mean of 544.3µm (±35.0µm) in the combined Australian (AU) twin cohort, and a mean of 545.8µm (±34.0 µm) in the UK twin cohort (Table 1). We report the results for standardized trait values – for effect sizes on the original scale, simply multiply by the trait standard deviation. We found weak association signals with the smallest p-values on the scales of 10^−6^ from the combined AU twin cohort alone, as was expected from the Q-Q plot (Figure S2). Except for one SNP suggesting a strong association signal (p-value of 2.9×10^−08^), the UK cohort was found mainly with weak associations (Figure S3). This may be due to the relatively small sample size of a single dataset, so we performed a first-stage meta-analysis on both twin cohorts as the Discovery sample. A common set of 524,813 SNPs was left after merging the datasets, among which 0.4% SNPs were further excluded because of ambiguous (A/T, C/G) polymorphism types at these loci. Furthermore, the effects and allele frequencies estimated from the UK samples were altered to have the same reference alleles as in the combined AU twin samples. The association signals were clearly enhanced in the meta-analysis. The meta-analysis results can be visualized by a Manhattan plot in Figure S4, and the top 5 SNPs with p-values from the association tests lower or around 1×10^−7^ are listed in Table 2.

**Table 1:** Descriptive statistics for central corneal thickness (CCT) in the three twin cohorts.

	AU twin cohort	UK twin cohort
Number of subjects	1714	1759
Number of families	786	1119
Mean age (sd)	21.4 (12.6)	54 (12)
Range of age	[5, 90]	[16, 82]
Sex (% Female)	56%	88.9%
Mean CCT	544.3	545.8
Standard deviation	35.0	34.0
Range of mean CCT	[381.5, 679.5]	[369, 657.5]

Data presented as a combined Australian (AU) twin cohort and a United Kingdom (UK) twin cohort. CCT is measured in µm. The CCT values are the mean CCT for both eyes (Materials and Methods). Full details of the AU and UK twin cohorts are given in Mackey et al [19] and Healey et al [20], respectively.

**Table 2:** Directly genotyped variants on meta-analysis of twin cohorts associated with central corneal thickness (CCT).

Marker	Chr	Coordinate (build36)	Nearest gene	Alleles^a^	AU Effect	(SE)	AU P	UK Effect	(SE)	UK P	Weighted Effect	(SE)	Meta-analysis P
rs12447690	16	86855625	*ZNF469*	T/C	0.122	0.04	3.66e-3	0.209	0.04	2.87e-8	0.170	0.03	1.67e-9
rs1006368	10	126336593	*FAM53B*	A/G	0.220	0.06	2.82e-4	0.225	0.06	4.23e-5	0.223	0.04	4.94e-8
rs11245330	10	126370382	*FAM53B*	A/G	0.220	0.06	2.87e-4	0.225	0.06	3.97e-5	0.223	0.04	4.94e-8
rs9938149	16	86889141	*ZNF469*	A/C	0.125	0.04	2.59e-3	0.210	0.05	5.32e-6	0.163	0.03	1.08e-7
rs2755237	13	40007429	*FOXO1*	A/C	0.200	0.06	3.24e-4	0.240	0.06	1.29e-4	0.217	0.04	1.57e-7

Top five SNPs associated with CCT in twin cohorts.

a The first letter listed in the column *Alleles* is the reference allele for the corresponding SNP, e.g., T is the reference allele for SNP rs12447690.

**Figure 1:**
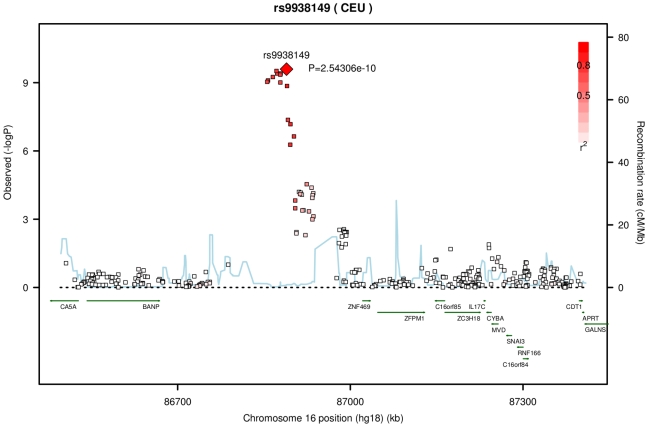
Significant association of central corneal thickness on chromosome 16 from the meta-analysis of the AU and UK twin cohorts. The top SNP rs9938149 had a p-value of 2.54×10^−10^ in the imputation set. The degree of linkage disequilibrium (LD) between rs9938149 and other SNPs is indicated by red shading. The recombination rate is displayed as a light blue line, with scale on the right hand axis. This imputed SNP is about 108 kb away from gene *ZNF469* (16q24, build 36.3), and there is evidence for recombination in the intervening region.

The results from meta-analysis of the twin cohorts revealed the most significant association around 86.86 Mb (build 36.3) on chromosome 16. The genotyped SNP rs12447690 had a strong genome-wide significant p-value of 1.67×10^−09^, with the stronger signal from the UK samples (Table 2). The fourth SNP in Table 2, rs9938149 is the top SNP in the imputation set (Figure 1). rs9938149 is 33.5 kb away from rs12447690, and they are in high linkage disequilibrium (LD) with r^2^ of 0.748. A few other imputed SNPs within 20 kb of the top SNP show strong association signals because of high LD in this region. The nearest gene is *ZNF469* on chromosome 16q24, 108 kb away from the top imputed SNPs. It is a small gene with a span of 42 kb (86,997K.87,039K, build 36.3). As shown in Figure 1, there is evidence for recombination between the associated SNPs and the putative gene *ZNF469*. This gene encodes a zinc-finger protein. Rare mutations in this gene cause Brittle Cornea Syndrome (BCS), a recessive disorder characterized by a thin cornea leading to progressive visual loss and blindness [13].

**Figure 2:**
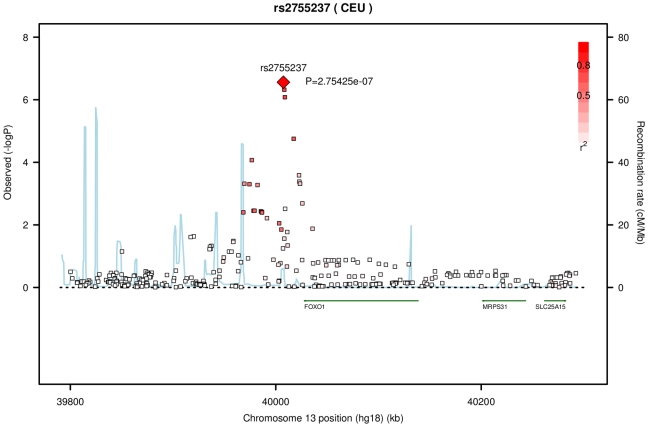
Association with CCT for variants on chromosome 13 from the meta-analysis of the AU and UK twin cohorts. The genotyped variant rs2755237 remained as the top SNP in the imputation set. It is about 20 kb from 3′ end of gene *FOXO1* (13q14.1, build 36.3). The degree of linkage disequilibrium between rs2755237 and other SNPs is indicated by red shading. The recombination rate is displayed as a light blue line, with scale on the right hand axis.

Another associated SNP in Table 2, rs2755237 (p = 1.57×10^−07^) is 20 kb from the 3′ end of the gene *FOXO1* on chromosome 13q14.1. It remained as the strongest signal in the imputation set (Figure 2). Two other SNPs approximately 1 kb away, rs2721051 and rs2755238, were masked by the top SNP in the plot. They are in high LD with rs2755237 (r^2^ of 0.736). The gene *FOXO1* belongs to the forkhead family of transcription factors, the same family as the candidate gene *FOXC1* (6q25) that was previously associated with early onset glaucoma (see Introduction).

The two remaining SNPs in Table 2 were found in the same region around 126,300K to126,400 K on chromosome 10. The SNPs rs1006368 and rs11245330 within the gene *FAM53B* (10q26.13), are 35kb apart and in complete LD (r^2^ of 1). They had the same meta-analysis p-value from the twin cohorts of 4.94×10^−08^. An imputed SNP rs4962399 also in this gene improved the p-value to 2.8×10^−09^ (Figure S5). Several other SNPs with a similar level of significance spread over this region also because of high LD. *FAM53B* was reported as related to the hypothetical protein LOC9679 in Reference Sequence database (RefSeq, NCBI).

### GWA results from population-based cohorts

**Figure 3:**
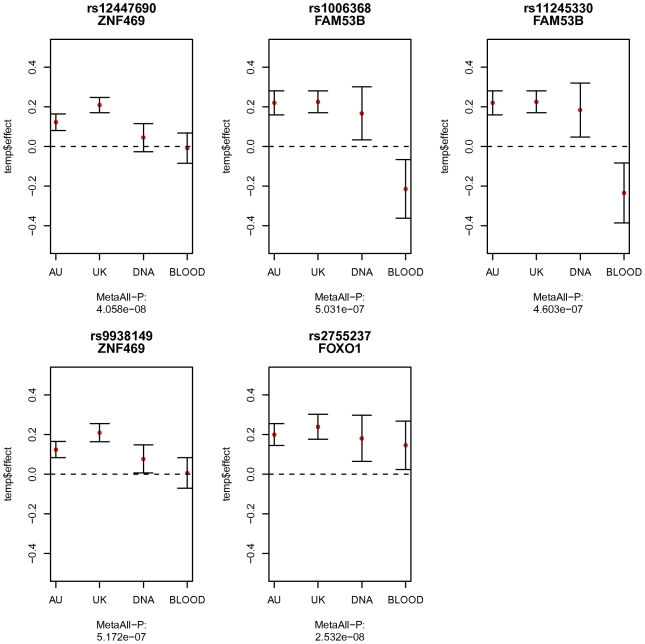
Comparison of the allelic effects estimated from the four cohorts. The four cohorts in comparison are the combined Australian twin cohorts (AU), the UK twin cohort (UK), the Blue Mountains population-based cohort in DNA pooling design (DNA), and the Adelaide population-based cohort in Blood pooling design (BLOOD). The mean allelic effect from each cohort is marked as red dot, with standard errors marked as bars. The overall meta-analysis p-value based on all the cohorts is shown under the x-axis label for each sub-figure. These SNPs are the most significant variants found in the meta-analysis of twin cohorts. The estimated effects from pooled samples of rs12447690 and rs9938149 around *ZNF469* region, were smaller than the effects in the twin cohorts. rs2755237 near *FOXO1* replicated well in pooled samples, with similar effects shown in all the cohorts. Two SNPs within gene *FAM53B* had an opposite direction of the effects found in the Blood pools.

As demonstrated above, evidence for association with CCT at loci on chromosomes 16, 13 and 10, was found in the Discovery sample of the meta-analyzed twin cohorts. As the replication, we conducted another GWA study on the population-based cohorts using pool genotyping design and validated the findings by individually genotyping the extended cohort (pooled samples plus extra samples with extreme phenotypes). Descriptive statistics of the two population-based cohorts are provided in Table 3. With the advantage of time and cost efficiency, pool genotyping design provided an expedient examination of the top variants from the results in the twin cohorts. We compared the allelic effect estimated from each cohort as shown in Figure 3. In order to reveal the association signals more clearly, we also performed a meta-analysis based on the two sets of GWA results from the twin samples and the population-based samples (results shown in Table S2, Manhattan plot in Figure S6).

**Table 3:** Descriptive statistics for central corneal thickness (CCT) in the two Australian pooled samples.

	Blue Mountains DNA pool	Adelaide Blood pool
Size of thin CCT group	143	106
Mean CCT (sd) in the case pool	495.52 (12.2)	488.1 (17.9)
Size of thick CCT group	146	105
Mean CCT (sd) in the control pool	584.83 (13.6)	600.1 (21.9)
Mean Age (years)	73.95	70.76
Sex (% Female)	57.3%	55. 5%

CCT is measured in µm. DNA or Blood samples from the thin CCT group were constructed as the case pool, and samples from the thick group as the control pool.

The most significant SNP rs12447690 from the twin cohorts had smaller effect sizes estimated from the pooled samples (Figure 3). Similar results were found in the other SNP rs9938149 in the *ZNF469* region (33.5kb away from the most associated SNP rs12447690). Although there was an overall genome-wide significant p-value for rs12447690 in the meta-analysis with twin cohorts (Table S2), the results from pooled samples alone did not show a clear replication for the SNPs near *ZNF469*. However, when we added the additional samples to the pooled samples, the individual genotyping results did show evidence for replication (p = 0.014) in the extended cohort (pooled+extra samples). The meta-analysis based on all the individually genotyped samples yielded an overall p-value of 8.95×10^−11^ for the SNP rs12447690. The final results summarizing the findings from all the individually genotyped samples are presented in Table 4.

**Table 4:** Association results for the three SNPs individually genotyped on all samples.

Marker	Chr	Coordinate (build36)	Nearest gene	Alleles^a^	Meta-analyzed Twin cohorts			Extended cohort (pool samples plus extra samples)			Overall Meta-analysis		
					Effect	(SE)	P	Effect	(SE)	P	Weighted Effect	(SE)	P
rs12447690	16	86855625	*ZNF469*	T/C	0.170	0.03	1.67e-9	0.140	0.06	0.014	0.162	0.03	8.95e-11
rs2721051	13	40008884	*FOXO1*	A/G	−0.221	0.04	4.87e-7	−0.303	0.08	2.7e-4	−0.240	0.04	4.60e-10
rs2755237	13	40007429	*FOXO1*	A/C	0.217	0.04	1.57e-7	0.175	0.07	0.013	0.206	0.04	7.02e-9

a The first letter listed in the column *Alleles* is the reference allele for the corresponding SNP.

In both DNA and blood pooled samples, SNP rs2755237 near *FOXO1* showed estimated allelic effects similar to the results from the twin cohorts (Figure 3). The other SNP rs2721051 in high LD with rs2755237 had a similar result (Table S2). This demonstrated a clear replication of these two variants from the pooled samples. The individual genotyped results on the extended cohort displayed the same pattern and obtained significances for both SNPs (Table 4). Due to the smaller pool sizes, the estimated allelic effects from the pooled samples have relatively larger standard errors and hence neither pool considered separately showed a significant (P<0.05) association (Table S2). In the overall meta-analysis, rs2721051 and rs2755237 obtained p-values of 4.6×10^−10^ and 7.02×10^−09^ respectively (Table 4).

The SNPs from the chromosome 10 region associated with CCT in the discovery sample had similar estimated effects in the first (DNA) pool. However effects in the opposite direction (e.g. negative effect for the same reference allele compared to positive effects in other samples) were observed in the second (blood) pool sample, indicating a possible false positive association with CCT (Figure 3).

Another interesting result arising from the meta-analysis of twin cohorts and the pooled samples is the SNP rs7044529, which is within the gene *COL5A1* (9q34.2–q34.3). As outlined in the introduction, the type V collagen gene *COL5A1* is a strong candidate for CCT, given the phenotypic association between the connective tissue disorder EDS and abnormal CCT values. Extremely thin corneas are common findings in EDS [9]. The estimated allelic effects for rs7044529 were similar in all the samples, with a weighted effect of −0.15. This SNP has a moderate overall p-value of 9.3×10^−06^ (Table S2).

## Discussion

CCT is an important clinical measurement of human eyes. Recent studies have highlighted CCT as a prognosticator for the development of glaucoma, one of the leading causes of irreversible blindness worldwide, with a thin CCT potentially increasing the risk of developing a subtype known as open-angle glaucoma (OAG) [2]–[4]. The genetic aetiology of OAG is not well understood, with only one major gene *myocilin* identified [14]. Given that OAG has a complex molecular aetiology, the breakdown of the dichotomous trait (i.e., “affected” or “unaffected” status) into its quantitative measurement will aid in the search for disease-susceptibility genes. It is therefore of highly clinical significance to explore the genetic factors that contribute to CCT variation. Thus, we conducted a multi-stage study on over 5,000 samples with the purpose of detecting the genetic variants for the human CCT. We conducted GWA studies on the discovery sample of the two twin cohorts from Australia and the UK. Another set of GWA studies were performed on the two population-based cohorts in pool genotyping design and the results were further validated by individual genotyping the extended cohort (pooled samples plus additional samples with extreme phenotypes).

We have identified a novel locus near *FOXO1* (overall p-value of 4.6×10^−10^ for SNP rs2721051), which accounts for ∼1.2% variation in normal human CCT. *FOXO1*, located at 13q14.1 is a 111kb gene belonging to the forkhead family of transcription factors and characterized by a distinct forkhead domain. Whilst the specific functions of this gene are unknown, it may play a role in myogenic growth and differentiation (RefSeq, NCBI). Translocation of this gene with *PAX3* has been associated with alveolar rhabdomyosarcoma (RefSeq, NCBI). A recent study by Berry et al. reported that the transcription factor gene *FOXC1* (6p25) regulates the expression of *FOXO1* and binds to a conserved element in the *FOXO1* promoter [15]. *FOXC1* is a major transcription factor involved in the development of the anterior segment of the eye, which is involved in both anterior segment dysgenesis and congenital glaucoma phenotypes [16].

In the twin cohorts we obtained genome-wide significant association for the genotyped SNPs rs12447690 (p = 1.67×10^−09^) and rs9938149 (p = 1.08×10^−07^), ∼140kb and ∼108kb respectively from the gene *ZNF469* (16q24). By individually genotyping the population-based samples with extreme CCT values, we showed that rs12447690 was well replicated with an overall p-value of 8.95×10^−11^, accounting for 1.29% of the variation in CCT. *ZNF469* was recently implicated in a study of the rare disorder BCS [13]. Abu et al. showed that rare sequence variants in *ZNF469* segregated with BCS. The SNPs we report near *ZNF469* have high MAFs – for example rs12447690 has MAF 0.44 in HapMap CEU samples, with a similar value in the cohorts presented here. Given the recombination hotspot (Figure 1) and the large difference in allele frequency between such variants and the rare variants identified by Abu et al., our findings are unlikely to be explained by linkage disequilibrium (LD) between the rare and common variants (the r^2^ parameter cannot be high between such polymorphisms). At the 16q24 locus there are four putative genes nearer to rs12447690 than *ZNF469*. However, in each case the putative genes are poorly characterized with only a hypothetical protein role.

Interestingly, one of the clinical features of BCS patients is hyperlaxity of the joints [13]. A small part of the AU twin cohort overlaps with samples from a pelvic floor study by Hansell et al [17], which included measurements of joint mobility [18] (Figure S7). Based on a small sample size of 102 individuals, CCT was inversely correlated (Pearson correlation −0.221, P = 0.02583) with thumb bending degree, but was uncorrelated with the other two measurements of joint mobility (Figure S7). To minimize multiple testing, we only tested for association of the thumb bending measure of joint mobility, and focused on 31 SNPs in the *ZNF469* region of interest. Despite the limited power in this study, 2 SNPs rs7198446 and rs7500421 in the underlying region were nominally associated with thumb bending degree, with p-values of 0.0298 and 0.0471 respectively (Figure S8). These SNPs are halfway (∼60kb to both sides) between the top SNPs on chromosome16 found in the CCT study and the gene *ZNF469*. We also checked the associations of these variants with CCT in this sample, but none of them was significant.

We have demonstrated a flexible approach to GWA studies using different designs. By taking into account of the thresholds used to determine high and low pools for the quantitative trait (together with population allele frequencies), we mapped the estimates of the differences in pooling allele frequency between high and low pools to the effect sizes on additive scale of the quantitative trait.

In summary, we identified a novel QTL for CCT near gene *FOXO1* (13q14.1) with p = 4.60×10^−10^. Common variants near *ZNF469* (16q24) were found in this study as associated with CCT with p = 8.95×10^−11^. Our findings suggest that in addition to rare variants in *ZNF469* underlying CCT variation in BCS patients, more common variants near this gene may contribute to CCT variation in the general population.

## Materials and Methods

### Ethics statement

This study was conducted according to the principles expressed in the Declaration of Helsinki. The study was approved by the human ethics committee of the University of Tasmania, Royal Victorian Eye and Ear Hospital, Queensland Institute of Medical Research and the Flinders Medical Centre. Informed consent was obtained from parents with the child's assent or from adult participants before testing.

### Twin cohorts

Three twin cohorts were recruited from Australia and the UK. The AU twin cohort consisted of two sub-samples, 953 individuals from the Brisbane Adolescent Twin Study (BATS) and 761 individuals from the Twin Eye Study in Tasmania (TEST), making up a whole cohort of 1714 participants from 786 families. A full description of the AU twin cohorts is given in Mackey et al [19]. Twins from the UK were a sub-sample from the cohorts collected at St Thomas' Hospital in London. 1759 people from 1119 families were included in this study. Nearly 90% of the UK samples are adult women. Details of the UK twin cohort are given in Healey et al [20]. CCT was measured in the twin cohorts using ultrasound pachymetry and recorded for both eyes. Measurements were performed using a Tomey SP 2000 (Tomey Corp., Nagoya, Japan) or a DGH Technology (model 500; Scarsdale, NY) pachymeter in the Australian and UK twin cohorts respectively. Twin pairs were measured at the same time of day to avoid bias related to diurnal variation. With little evidence for a significant difference between the left and right eyes (ANOVA p-value = 0.575), the mean CCT value of both eyes was used throughout as our measurement.

In the AU twin cohorts, DNA samples extracted from each person were hybridized to the Illumina HumanHap 610W Quad arrays, with the samples from BATS genotyped by deCODE Genetics and the ones from TEST genotyped by the Center for Inherited Disease Research (CIDR). We scrutinized the genotypic data (SNPs) and screened them according to a series of quality control criteria, including minor allele frequency (MAF)≥1%, p-value for Hardy-Weinberg equilibrium test≥10^−6^, SNP call rate>95% or Illumina Beadstudio GenCall score≥0.7. After cleaning, 530,656 SNPs were left for association testing in AU twin cohorts. The UK samples were partly genotyped on the Illumina Hap610W arrays at CIDR, and partly genotyped on Illumina HumHap 300K Duo arrays at Wellcome Trust Sanger Institute. Slightly different quality control criteria compared with the AU twin study were applied: MAF≥1%, p-value for Hardy-Weinberg equilibrium test≥10^−4^ and SNP call rate>95%, resulting in a complete set of 548,001 SNPs for the association tests.

We screened the genotypic data for ancestral outliers using principal component analysis [21]. By comparing AU twin data with 16 global populations sourced from HapMap Phase 3 and Northern European sub-populations from a previous study by McEvoy [22], 2% of the samples were excluded for being identified as ancestral outliers; thus giving us greater confidence in the homogeneity of the study sample (Figure S2). UK twin samples were also screened for genetic outliers by comparison with the reference of three main populations from HapMap Phase 2. The Q-Q plot (Figure S3) clearly shows the homogeneity of the UK panel except for one data point. The discrepancy between the observed and expected statistics for this variant suggests a potential association signal.

Higher density markers on autosomes were also available from imputation. We imputed data using MACH for the AU samples based on a set of 469,117 SNPs which were common to the six Illumina 610K subsamples at QIMR. The imputation for the UK samples were undertaken with reference to HapMap release 22 CEU using IMPUTE version 2 [23]. Each of the imputed datasets contains up to 2.4 million SNPs.

Both AU and UK twin cohorts in our study consist of either twin pairs or their close relatives (parents, siblings) in the family. Samples within the family are genetically related, sharing the chromosomal regions of identity-by-descent (IBD). In those regions, the related samples will provide the similar genetic information. Failing to estimate the IBD states will result in an increased false-positive rate in the association tests. To avoid this problem, we conducted the association test (–fastassoc) in MERLIN [24]. It incorporated genetic relatedness between the samples by estimating the IBD prior to the association tests. The AU samples were controlled for both age and gender effects, whilst the predominantly female UK samples were only controlled for age effects. We standardized the trait distribution of CCT to increase the inter-sample compatibility as well as robustness to extreme observations.

### Population-based cohorts

Two population-based cohorts were studied in the case-control pool design. We measured and recorded CCT for both cohorts in the same way as in the Australian twin cohorts.

The first cohort utilized was the Blue Mountains Eye Study (BMES). This population-based study, designed to investigate the genetics and epidemiology of ocular disease, recruited 3654 individuals living in a defined geographical region in the Blue Mountains (west of Sydney, Australia). Both DNA and CCT measurements were available for 953 individuals. Jawaid et al showed that the optimal fraction for quantitative trait locus (QTL) mapping using pooled DNA samples was 20% [25]. Thus in this study, DNA samples extracted from the individuals among the thick CCT group (upper 20% of the CCT distribution) were constructed as a control pool, whereas DNA samples from the thin group (lower 20% of the distribution) as a case pool. This resulted in 190 individuals in each tail although in practice sufficient DNA was only available for 143 individuals in the thin pool and 146 individuals in the thick pool. The drop-out due to insufficient DNA was random with respect to phenotype, suggesting we were effectively sampling the extremes from a total sample size of ∼145/190 * 953 = 727. Concentration of DNA samples were carefully adjusted by serial dilutions and quantified using PicoGreen (InVitrogen), to ensure the equal quantity of DNA contributed by individual samples. Pooled DNA was genotyped on Illumina Human 1M-Duo V3 arrays at Queensland Institute of Medical Research (QIMR, Brisbane, Australia) in triplicate.

The second cohort, based on a blood pooling design [26] was collected from Adelaide, Australia. This study consisted of 530 unrelated individuals in total, with 106 individuals in the thin CCT group (covers 20% lower tail of the underlying CCT distribution) and 105 individuals in the thick CCT group (covers 20% upper tail). The CCT values for the middle group were not recorded. Equal quantity (100 µL) of whole blood was aliquoted shortly after venesection from each individual. This aliquot was stored at 4°C then lysed immediately prior to pooling. A single DNA extraction was then performed on each blood pool using QIAmp maxi kit (Qiagen). Each blood pool was genotyped on Illumina Human 1M-Duo V3 arrays at QIMR, with four replications.

The output of the raw red and green bead scores from the genotyping stage was available for the pooled data analysis. We applied the same data processing protocol to both cohorts, similar to the method described in the supplementary methods in Brown et al [27]. Before calibrating the raw scores, a number of SNPs with more than 10% negative scores on each array were excluded, as well as the SNPs with the sum of mean red and green scores across each array smaller than 1200. This step was included to ensure that the calibration was done on a pre-cleaned dataset. A normalization/correction factor (*corr*) was calculated by forcing the mean value of the pooling allele frequency to be 0.5 over all SNPs on each stripe (Illumina Human 1M-Duo V3 array has 6 stripes on a single array). The pooling allele frequency (PAF) was then estimated based on the raw red intensities and the corrected green intensities for all the SNPs (PAF = red/(red+green/*corr*)).

A final set of autosomal SNPs met the following criteria: more than 5 probes in each pool; with a MAF greater than 1%; without a significant variance difference between case and control pools (i.e., the log_10_ transformed p-values from an F test on the ratio of case control pool variances were smaller than 6), was taken forward to a linear regression model [28]. The underlying idea was to regress the pooling allele frequency on the case/control status for each SNP and estimate the pooling error across all the SNPs (for more details see MacGregor et al [28], [29]). The p-value from comparing the test statistic in the MacGregor paper (T_2-x_) to χ^2^_(1)_ distribution was computed to assess the significance of allele frequency difference between the two pools (*d*).

Individual SNPs of interest were genotyped in most individuals included in the DNA and blood pools as well as additional 102 samples (72 samples from BMES population and 30 samples from Adelaide population) belonging to the extreme quantiles of the CCT distribution but not available for pooling. SNPs were genotyped using iPLEX GOLD chemistry (Sequenom) on an Autoflex Mass Spectrometer (Sequenom) at the Australia Genome Research Facility (Brisbane, Australia).

### Method to compare association results in different designs

Since the pooling design dichotomizes the quantitative trait of CCT as a binary trait (case/control status), results from the pooling cohorts are not comparable with those from the twin cohorts. An alternative way of enabling such comparison is to transform the case/control frequency difference (*d*) to be the allelic effect (*β*), given information on the allele frequency (*p*) and the upper/lower threshold cutting up both tails (*T_U_/T_L_*). Following the notions in Jawaid et al [25], the expected allele frequencies in the two pools are
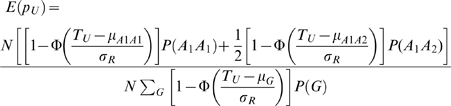


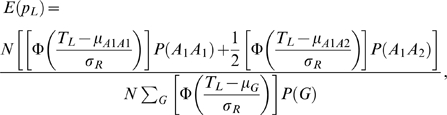
where N is the sample size; Φ is the density function of standard normal distribution; T_U_ and T_L_ are the upper and lower thresholds; P(G) is the genotypic frequency; μ_G_ stands for the mean trait value for the corresponding genotype; 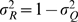, assume no dominance effects for the QTL, then 
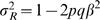
 is the trait variance for each genotype. Thus the case control frequency difference between the two pools is 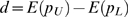. It demonstrates the relation between the case control frequency difference (*d*) in a pooling design and the allelic effect (*β*) in a conventional design, given the allele frequency (*p*) and the upper/lower threshold (*T_U_/T_L_*). Based on the inverse function of *d*, the allelic effect can be obtained from the estimated frequency difference of the case control pools.

As described earlier, the lower threshold in this study is the 20% quantile of the standard normal distribution and the 80% quantile for the upper threshold. The allele frequencies estimated from the combined AU twin cohort were fitted as the allele frequency parameter, *p* in this context.

We also applied the association mapping method for selective genotyping design in Huang and Lin [30] to the combined cohort with extreme CCT phenotypes (pooled samples plus extra samples which were individually genotyped for a small number of SNPs). Our settings fit in the second design in their paper, namely, a random sample of n individuals whose trait values fall into certain regions is selected for genotyping and the trait values are retained for only those individuals. Therefore, we utilized the conditional likelihood described in the paper to obtain the unbiased estimator of the effect size and its standard deviation.

### Meta-analysis method

As mentioned above, the mean CCT values were measured and standardized in the same way for all the five cohorts. Since each of the sample size with all the Caucasian samples was sufficiently large, the distributions of CCT values in all the cohorts were good approximations of CCT distribution for Caucasian population found in clinical studies, normal distribution with mean ∼540µm [31] (Figure S9). These compatibilities ensured the comparison of the results from two or more cohorts in a single meta-analysis. It will considerably enlarge the overall sample size and increase the power to identify associations. A test statistic for the meta-analysis,
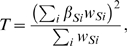
with *β* as the allelic effect from sample *Si* and the weight 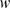 as its inverse variance, is expected to be distributed as a chi-square with 1 degree of freedom. This test assessing the significance of the weighted effect size with respect to its combined variance, has the advantage of taking into account the direction of the allelic effect. Therefore, the reference alleles from all the samples were required to be coordinated before the meta-analysis. In our study, a small proportion (<1%) of SNPs with the ambiguous polymorphism types (A/T, C/G) were excluded prior to our main analyses.

## Supporting Information

Figure S1 Schematic of the study design. The whole study is divided into three phases. Phase I, we conducted genome-wide association (GWA) studies on the two twin cohorts from Australia (T1) and the UK (T2) separately. The first stage meta-analysis on the twin cohorts uncovered three chromosomal regions showing evidence for association with CCT. Phase II, we conducted another set of GWA studies on the two population-based cohorts using pool genotyping design (P1, P2), which allowed the quick examination of the variants from Phase I. We also performed the meta-analysis on the two sets of GWA results (denoted by I+II). Phase III, the SNPs of interest from Phase II were further validated by individually genotyping the extended cohort (pooled samples P1, P2 plus extra samples P3). The final results of three associated SNPs in two regions were provided in an overall meta-analysis based on all the individual genotyped samples (denoted by I+III). For sample information, refer to Materials and Methods and Table S1.
(0.43 MB TIF)

Figure S2 Q-Q plot for the Australian (AU) twin cohort. The general concordance between the observed and the expected chi-square statistics indicates the homogeneity of the samples. The top data points within the shade zone (confidence interval) shows no evidence for strong association in the AU data alone.
(0.16 MB TIF)

Figure S3 Q-Q plot for the UK twin cohort. Nearly all the data points are within the shade zone (confidence interval) except the top one suggesting a potential strong association signal.
(0.17 MB TIF)

Figure S4 Manhattan plot for the meta-analysis of GWA results from both twin samples.
(0.15 MB TIF)

Figure S5 Association of central corneal thickness with variants on chromosome 10 from the meta-analysis of the AU and UK twin cohorts. The top imputed SNP rs4962399 was within the gene *FAM53B* (10q26.13). Several SNPs spread over this region (around 126,300K to 126,500K) with similar significance levels were due to high LD, which is indicated by red shading. The recombination rate is displayed as a light blue line, with its scale on the right hand axis.
(0.25 MB TIF)

Figure S6 Manhattan plot for the meta-analysis of GWA results from the twin samples and the pooled samples.
(0.15 MB TIF)

Figure S7 Three measurements of joint mobility described in Simpson [18]. In this study, the measurements were recorded as follows, and the phenotypic data were analyzed as a quantitative trait in a scale by degree: (A) The degree of apposition of the thumb to the flexor aspect of the forearm; (B) The degree of passive dorsiflexion of the metacarpophalangeal joint; (C) The degree of hyperextension of the elbow.
(0.92 MB TIF)

Figure S8 Nominally significant variants for the phenotype of thumb bending degree. The significant variants were found in the *ZNF469* region given a small sample size (n = 102). The SNPs rs7198446 (p = 0.02977) and rs7500421 (p = 0.0471) were in linkage disequilibrium (r2) of 0.17. These SNPs were in between (∼60kb to both sides) the top SNPs on chr16 from central corneal thickness study and the gene *ZNF469*.
(0.23 MB TIF)

Figure S9 Distributions of central corneal thickness. The central corneal thickness (CCT) distributions were presented for three cohorts: the combined Australian twin cohort (AUtwin), the UK twin cohort (UKtwin), the Blue Mountains population-based cohort in DNA pooling design (samples in DNA pool). Since the samples with non-extreme CCT values in Blood pooling design were not recorded, its distribution was not presented. However, by comparing the tails from samples in Blood pooling design with the ones in DNA pooling design we know that the whole distribution should be same with other cohorts, normally distributed with mean of ∼540 µm.
(0.25 MB TIF)

Table S1 Summary of the sample sizes of the study populations.
(0.01 MB PDF)

Table S2 Top genotyped variants from the association test based on the meta-analysis of two twin samples and two pooled samples.
(0.03 MB PDF)
