# Supplementary figures and images for: Common Genetic Variants near the Brittle Cornea Syndrome Locus ZNF469 Influence the Blinding Disease Risk Factor Central Corneal Thickness
Source: PLoS Genet. 2010 May 13;6(5):e1000947. doi: 10.1371/journal.pgen.1000947 (PMC2869325; doi:10.1371/journal.pgen.1000947)

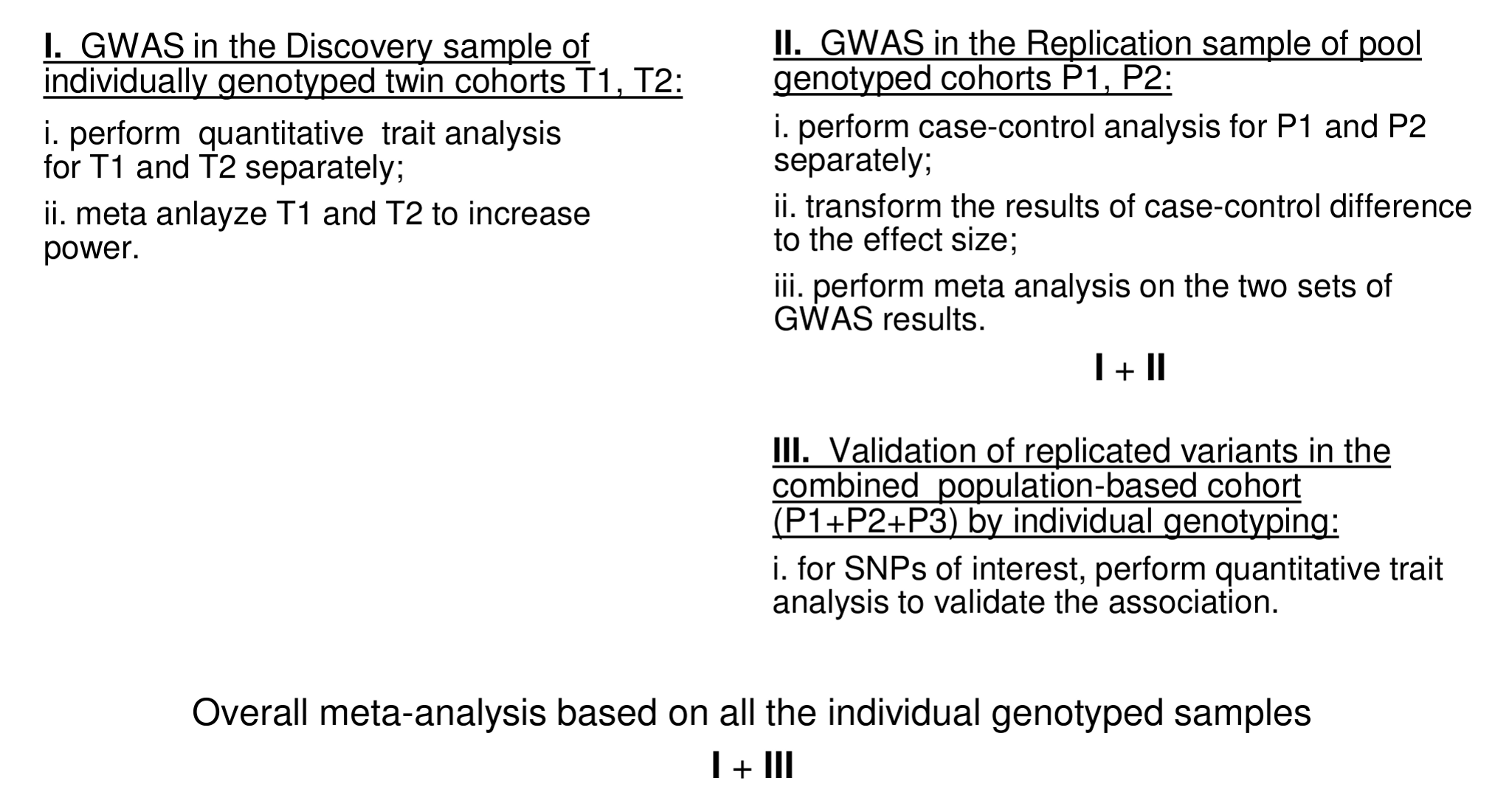

Supplement: Figure S1 — Schematic of the study design. The whole study is divided into three phases. Phase I, we conducted genome-wide association (GWA) studies on the two twin cohorts from Australia (T1) and the UK (T2) separately. The first stage meta-analysis on the twin cohorts uncovered three chromosomal regions showing evidence for association with CCT. Phase II, we conducted another set of GWA studies on the two population-based cohorts using pool genotyping design (P1, P2), which allowed the quick examination of the variants from Phase I. We also performed the meta-analysis on the two sets of GWA results (denoted by I+II). Phase III, the SNPs of interest from Phase II were further validated by individually genotyping the extended cohort (pooled samples P1, P2 plus extra samples P3). The final results of three associated SNPs in two regions were provided in an overall meta-analysis based on all the individual genotyped samples (denoted by I+III). For sample information, refer to Materials and Methods and Table S1. (0.43 MB TIF) [file pgen.1000947.s001.tif]

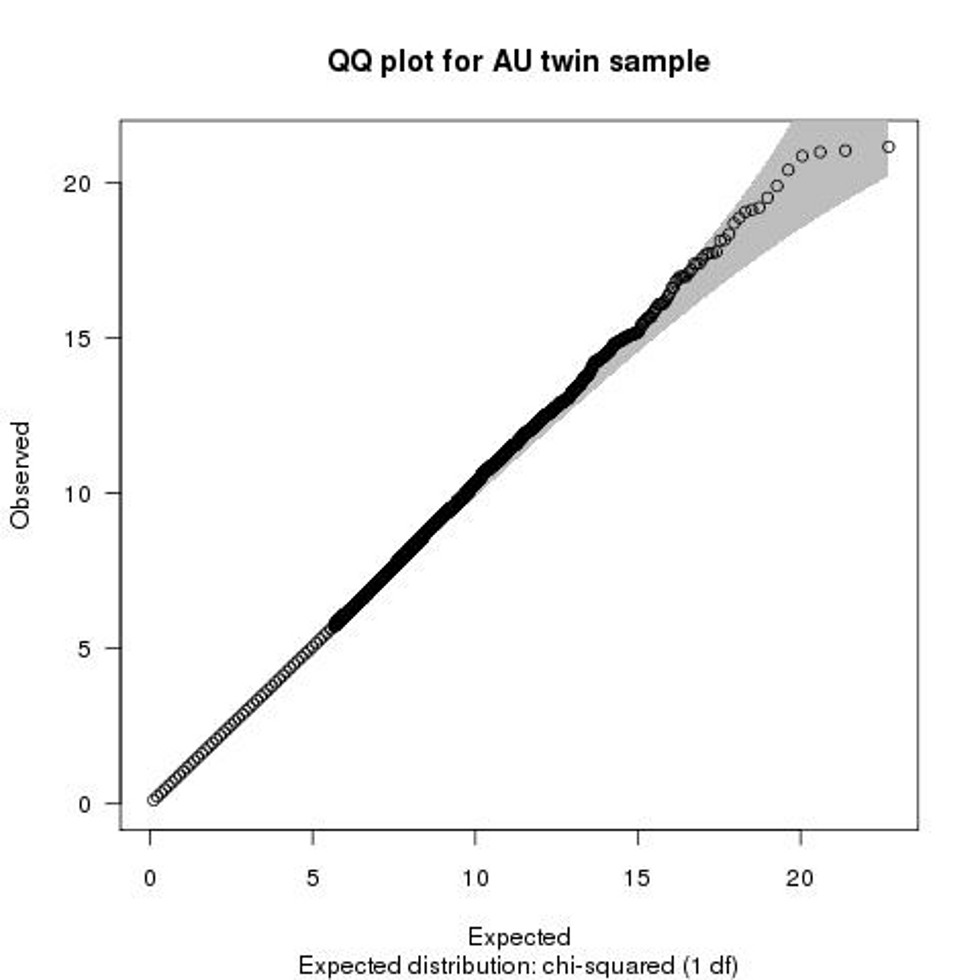

Supplement: Figure S2 — Q-Q plot for the Australian (AU) twin cohort. The general concordance between the observed and the expected chi-square statistics indicates the homogeneity of the samples. The top data points within the shade zone (confidence interval) shows no evidence for strong association in the AU data alone. (0.16 MB TIF) [file pgen.1000947.s002.tif]

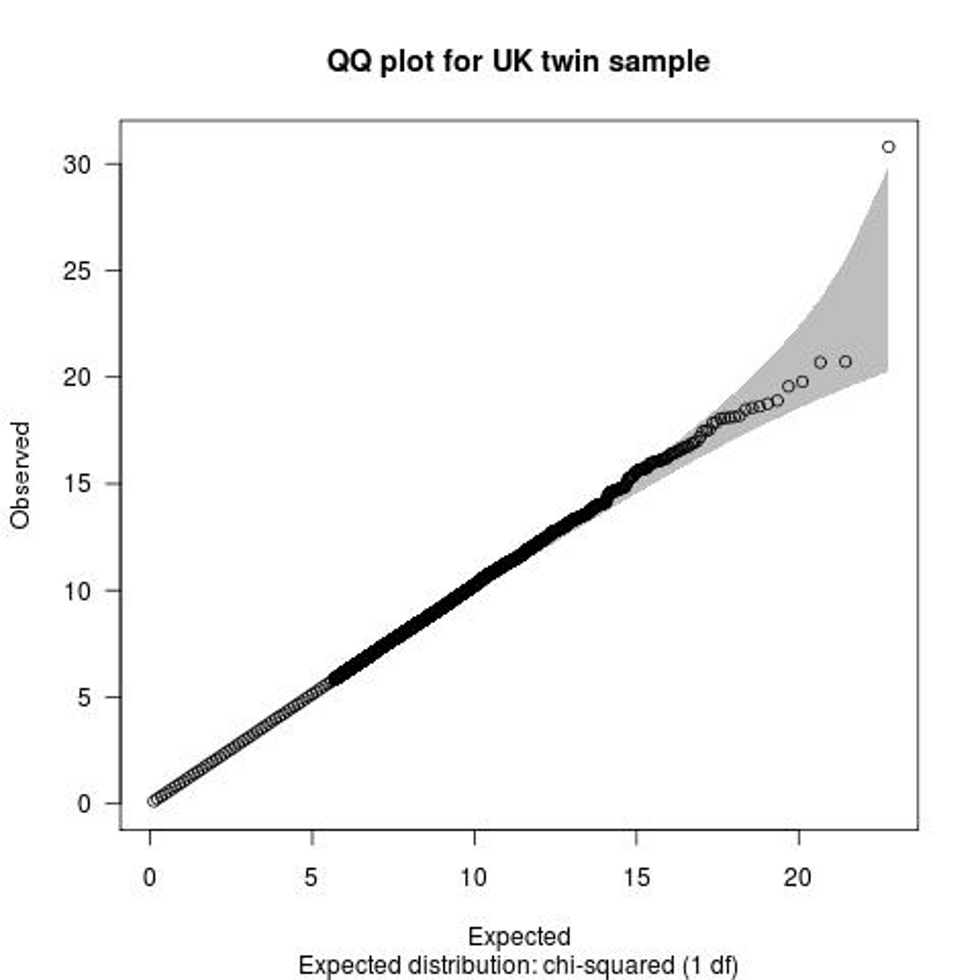

Supplement: Figure S3 — Q-Q plot for the UK twin cohort. Nearly all the data points are within the shade zone (confidence interval) except the top one suggesting a potential strong association signal. (0.17 MB TIF) [file pgen.1000947.s003.tif]

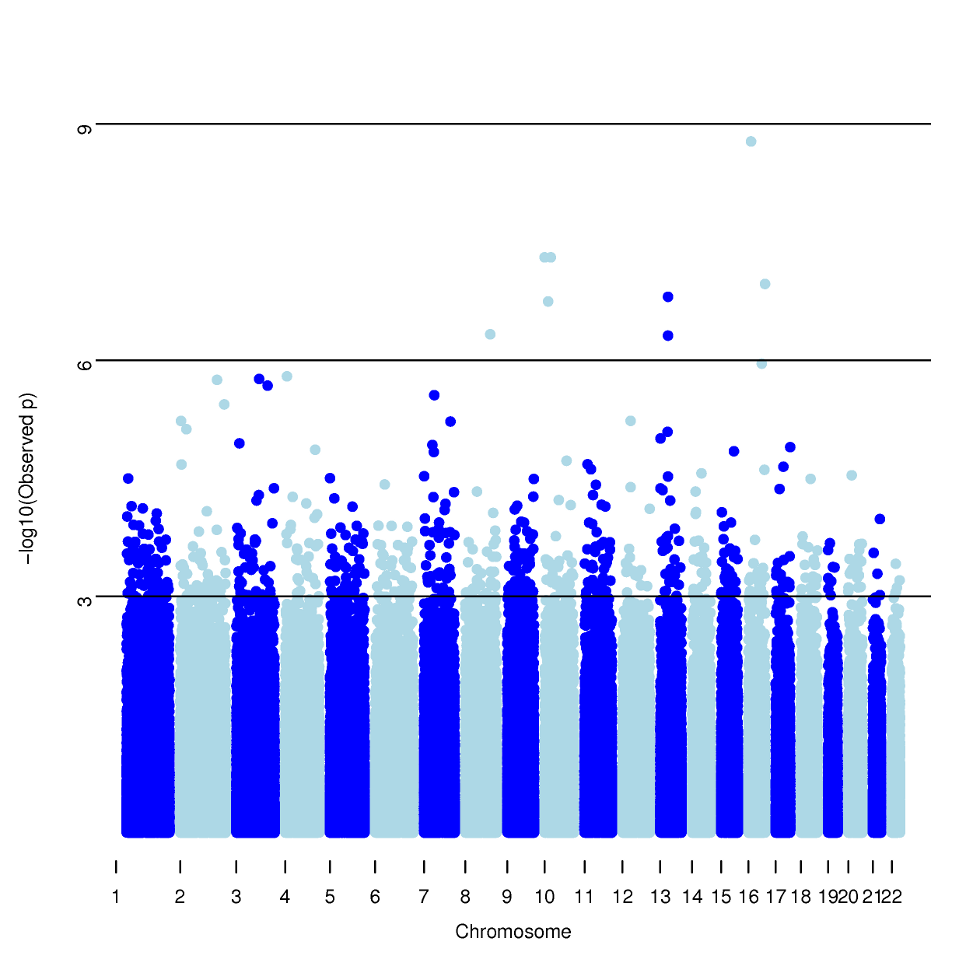

Supplement: Figure S4 — Manhattan plot for the meta-analysis of GWA results from both twin samples. (0.15 MB TIF) [file pgen.1000947.s004.tif]

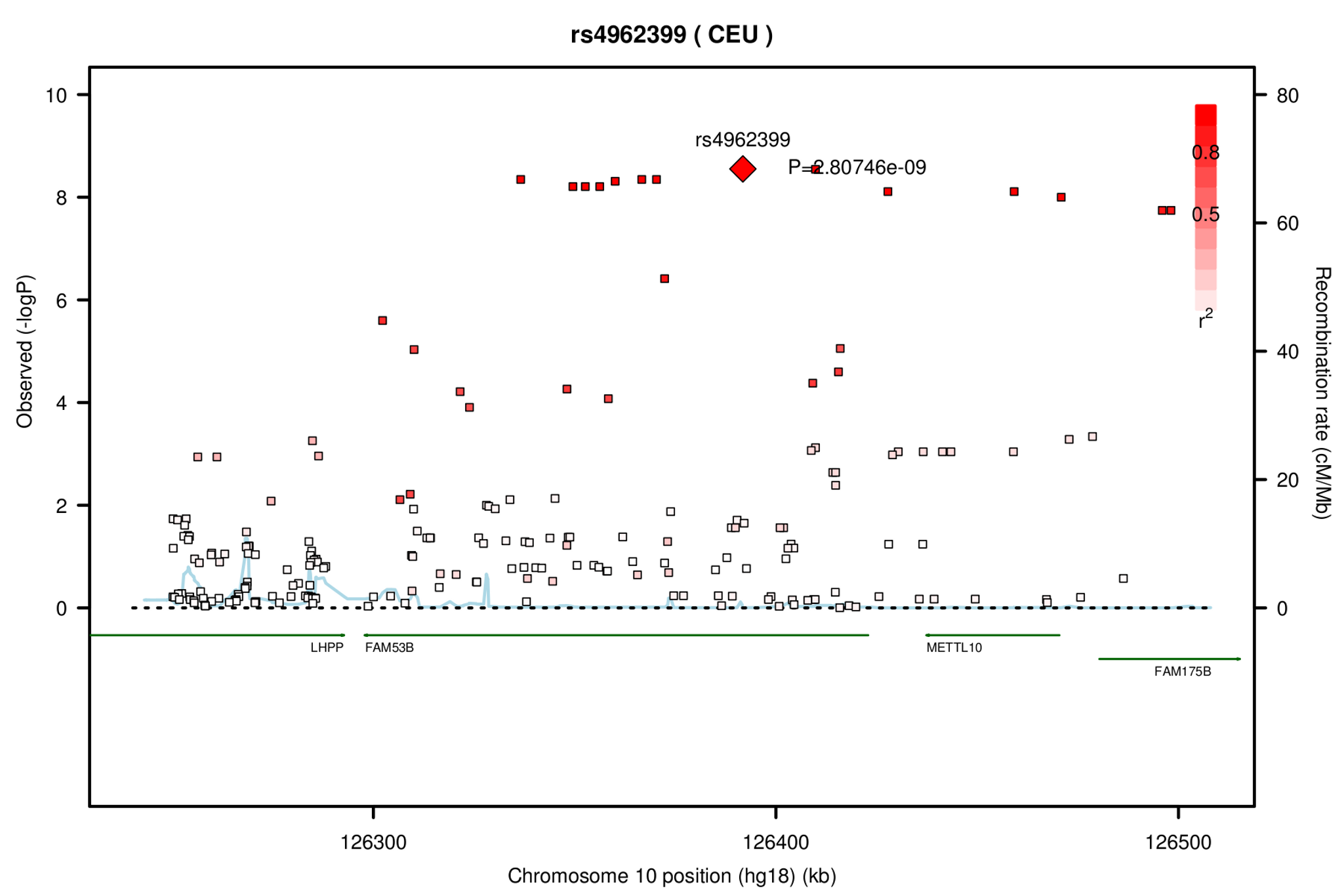

Supplement: Figure S5 — Association of central corneal thickness with variants on chromosome 10 from the meta-analysis of the AU and UK twin cohorts. The top imputed SNP rs4962399 was within the gene FAM53B (10q26.13). Several SNPs spread over this region (around 126,300K to 126,500K) with similar significance levels were due to high LD, which is indicated by red shading. The recombination rate is displayed as a light blue line, with its scale on the right hand axis. (0.25 MB TIF) [file pgen.1000947.s005.tif]

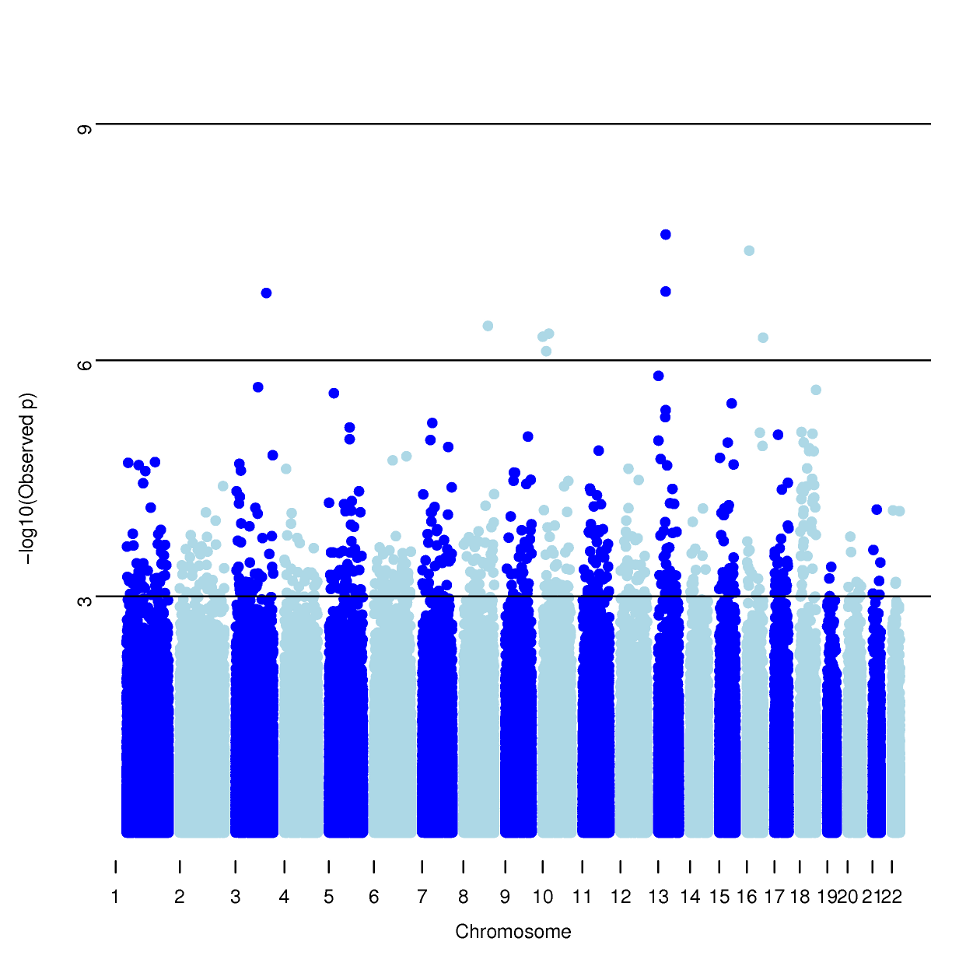

Supplement: Figure S6 — Manhattan plot for the meta-analysis of GWA results from the twin samples and the pooled samples. (0.15 MB TIF) [file pgen.1000947.s006.tif]

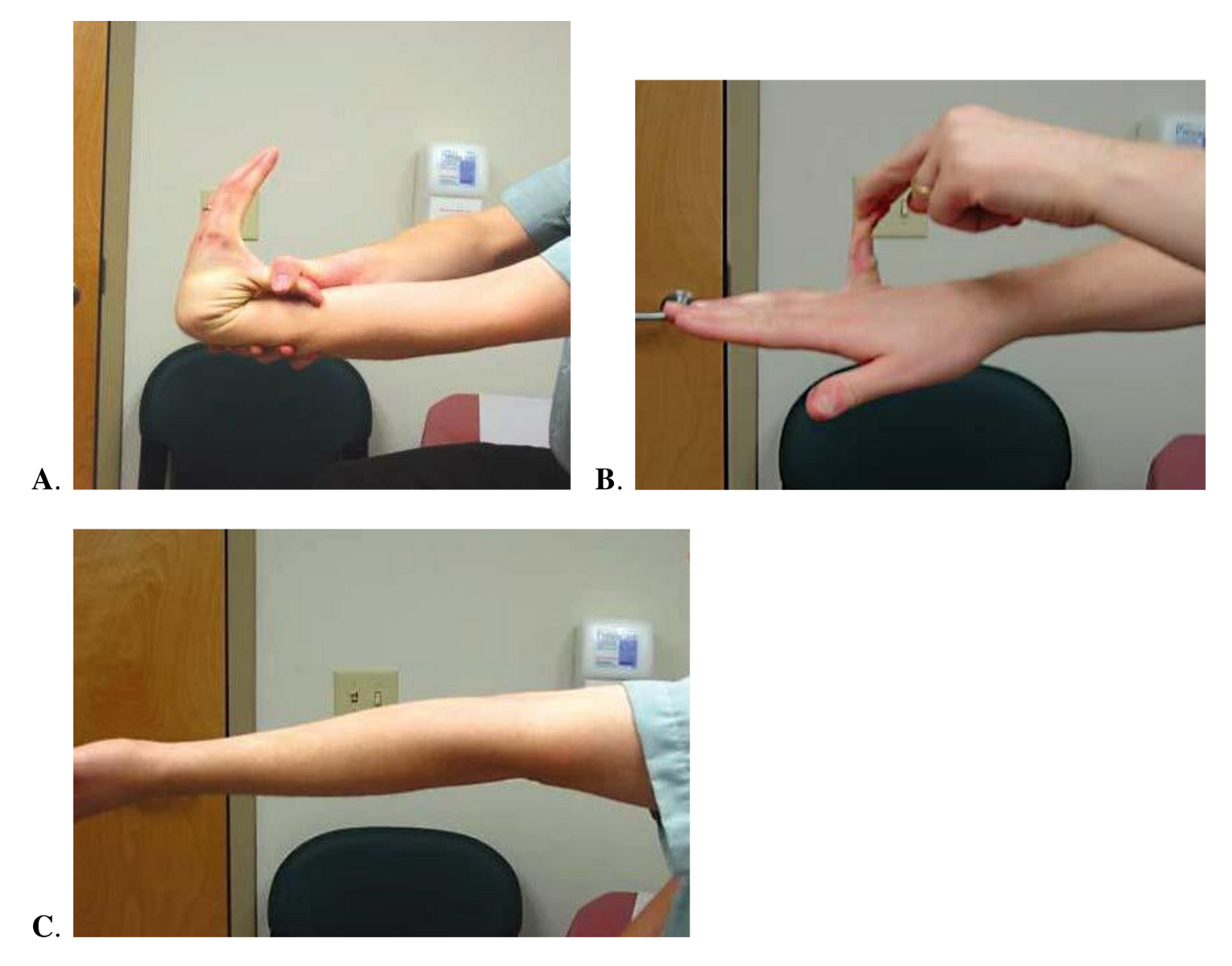

Supplement: Figure S7 — Three measurements of joint mobility described in Simpson [18]. In this study, the measurements were recorded as follows, and the phenotypic data were analyzed as a quantitative trait in a scale by degree: (A) The degree of apposition of the thumb to the flexor aspect of the forearm; (B) The degree of passive dorsiflexion of the metacarpophalangeal joint; (C) The degree of hyperextension of the elbow. (0.92 MB TIF) [file pgen.1000947.s007.tif]

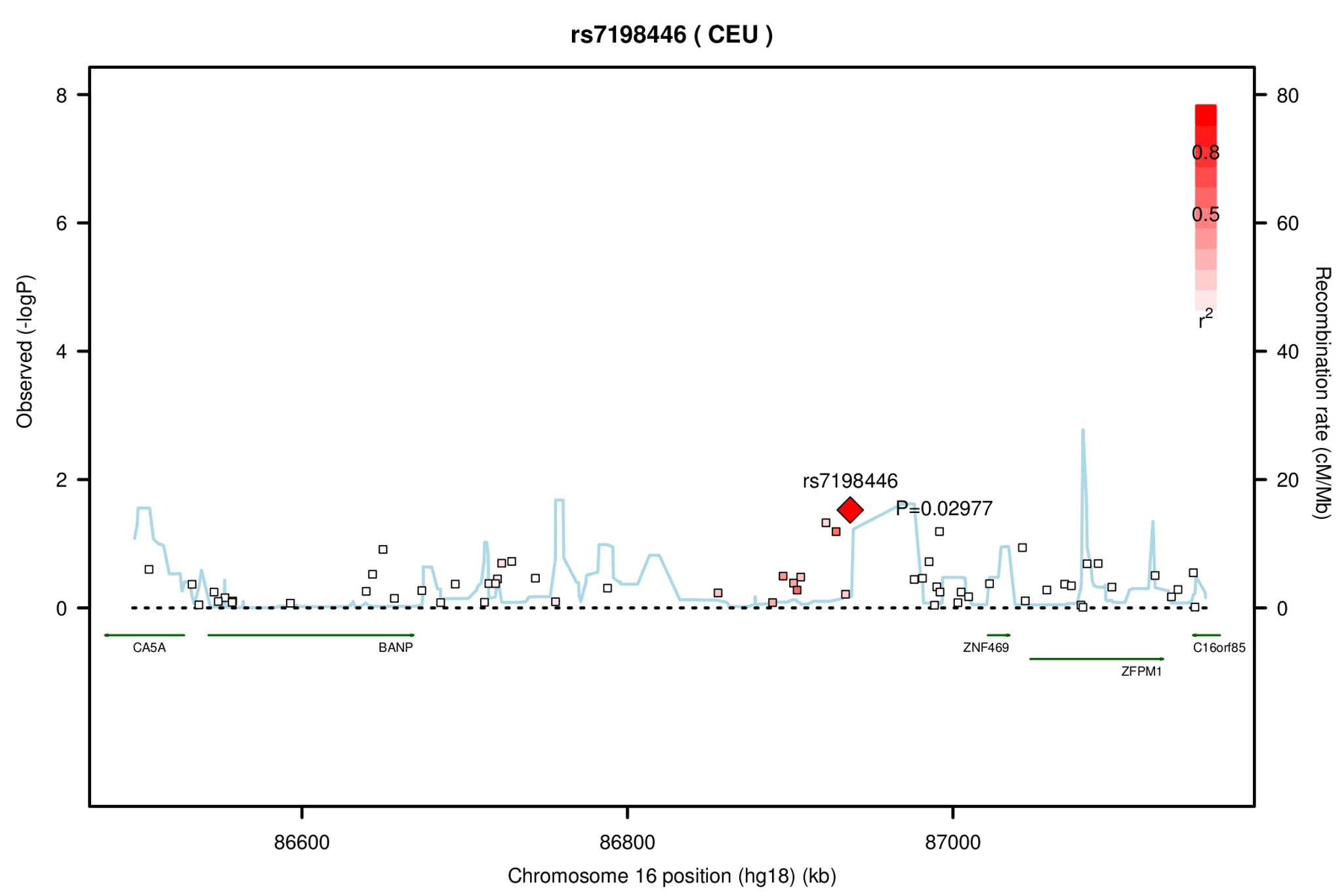

Supplement: Figure S8 — Nominally significant variants for the phenotype of thumb bending degree. The significant variants were found in the ZNF469 region given a small sample size (n = 102). The SNPs rs7198446 (p = 0.02977) and rs7500421 (p = 0.0471) were in linkage disequilibrium (r2) of 0.17. These SNPs were in between (∼60kb to both sides) the top SNPs on chr16 from central corneal thickness study and the gene ZNF469. (0.23 MB TIF) [file pgen.1000947.s008.tif]

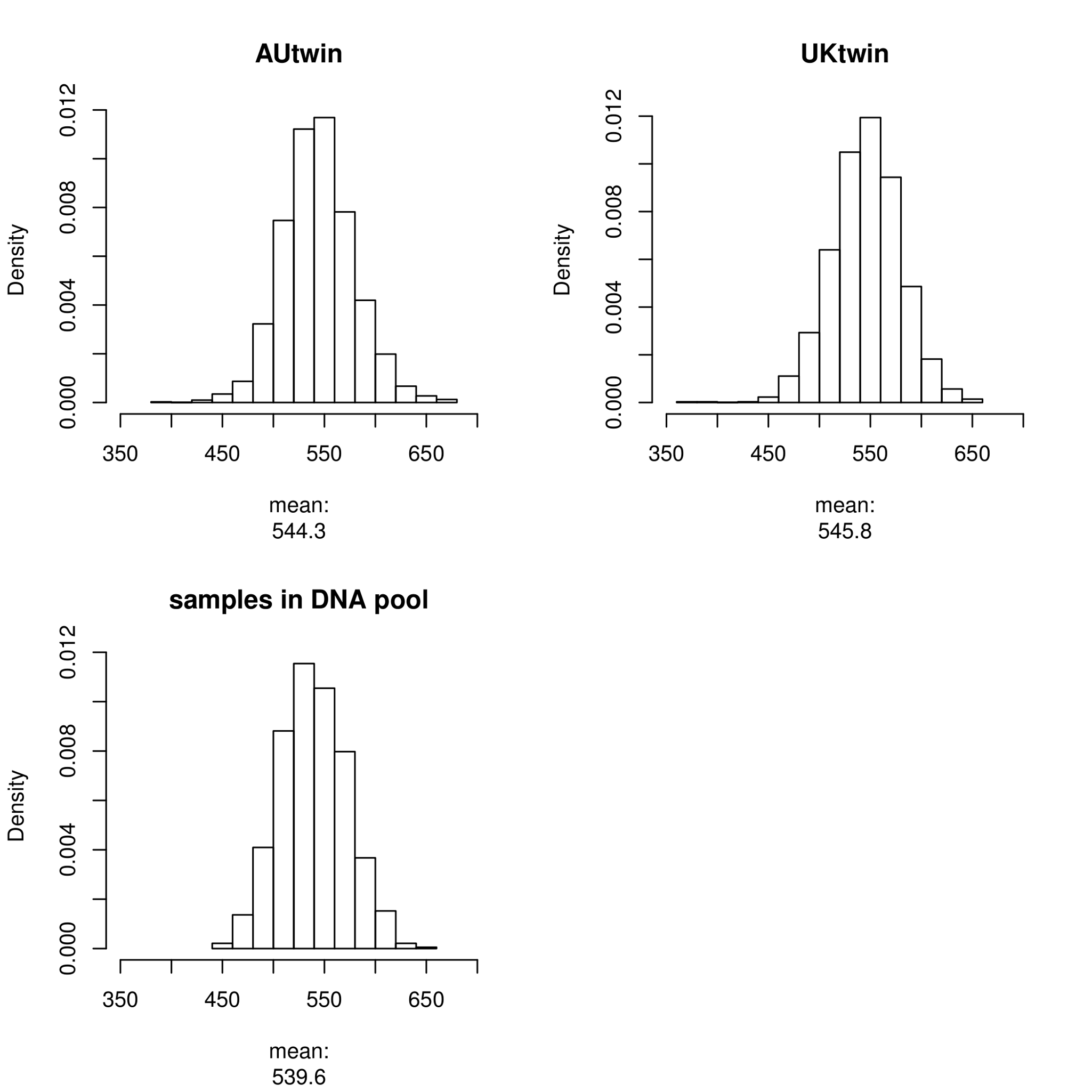

Supplement: Figure S9 — Distributions of central corneal thickness. The central corneal thickness (CCT) distributions were presented for three cohorts: the combined Australian twin cohort (AUtwin), the UK twin cohort (UKtwin), the Blue Mountains population-based cohort in DNA pooling design (samples in DNA pool). Since the samples with non-extreme CCT values in Blood pooling design were not recorded, its distribution was not presented. However, by comparing the tails from samples in Blood pooling design with the ones in DNA pooling design we know that the whole distribution should be same with other cohorts, normally distributed with mean of ∼540 µm. (0.25 MB TIF) [file pgen.1000947.s009.tif]
