# Supplementary material for: Common Genetic Variants near the Brittle Cornea Syndrome Locus ZNF469 Influence the Blinding Disease Risk Factor Central Corneal Thickness
Source: PLoS Genet. 2010 May 13;6(5):e1000947. doi: 10.1371/journal.pgen.1000947 (PMC2869325; doi:10.1371/journal.pgen.1000947)
Supplement: Table S1 — Summary of the sample sizes of the study populations. (0.01 MB PDF) [file pgen.1000947.s010.pdf]

Table S1. Summary of the sample sizes of the study populations.

Study population	Quantitative trait analysis	Case-control analysis	
	Total sample size	Case (lower 20%)	Control (upper 20%)
AU twin (T1)[a]	1714		
UK twin (T2)	1759		
BMES population (P1)		143	146
Adelaide population (P2)		106	105
Additional AU population (P3)[b]		52	50
Extended AU population (P1+P2+P3)[c]	587		

a: Australian twin cohort (T1) is a combined cohort of two sub-samples, 953 individuals from the Brisbane Adolescent Twin Study (BATS) and 761 individuals from the Twin Eye Study in Tasmania (TEST).
b: The additional AU population contains 102 extra samples: 72 samples from BMES population but not included in the DNA pools and 30 samples from Adelaide population but not included in the blood pools.
c: The extended AU population mainly consists of the BMES population (P1), Adelaide population (P2) and the extra samples from these two populations (P3). However, due to insufficient DNA stocks being available for some samples, 15 samples from the BMES population were included in the DNA pools but not individually genotyped.
