# Supplementary material for: Common Genetic Variants near the Brittle Cornea Syndrome Locus ZNF469 Influence the Blinding Disease Risk Factor Central Corneal Thickness
Source: PLoS Genet. 2010 May 13;6(5):e1000947. doi: 10.1371/journal.pgen.1000947 (PMC2869325; doi:10.1371/journal.pgen.1000947)
Supplement: Table S2 — Top genotyped variants from the association test based on the meta-analysis of two twin samples and two pooled samples. (0.03 MB PDF) [file pgen.1000947.s011.pdf]

Table S2. Top genotyped variants from the association test based on meta-analysis of two twin samples and two pooled samples.

Name	Chr	BP	gene	Alleles	AU twin sample		UK twin sample		DNA Pool sample		Blood Pool sample		Meta of all samples	
					Effect (sd)	pval	Effect (sd)	pval	Effect (sd)	pval	Effect (sd)	pval	Effect (sd)	pval
rs2755237	13	40007429	FOXO1	A/C	0.20 (0.06)	3.2E-04	0.24 (0.06)	1.3E-04	0.18 (0.12)	0.12	0.15 (0.12)	0.23	0.21 (0.04)	2.5E-08
rs12447690	16	86855625	ZNF469	T/C	0.12 (0.04)	3.7E-03	0.21 (0.04)	2.9E-08	0.05 (0.07)	0.52	-0.01 (0.08)	0.92	0.14 (0.02)	4.1E-08
rs2721051	13	40008884	FOXO1	A/G	-0.25 (0.07)	1.3E-04	-0.20 (0.06)	9.9E-04	-0.23 (0.16)	0.16	-0.15 (0.18)	0.40	-0.22 (0.04)	1.3E-07
rs7643363	3	173398470	FNDC3B	T/C	-0.14 (0.06)	1.3E-02	-0.30 (0.07)	9.7E-06	-0.14 (0.13)	0.27	-0.26 (0.12)	0.03	-0.21 (0.04)	1.4E-07
rs6990252	8	39935625	INDOL1	A/G	0.14 (0.05)	5.4E-03	0.19 (0.05)	1.6E-05	0.09 (0.09)	0.37	0.09 (0.11)	0.41	0.15 (0.03)	3.7E-07
rs11245330	10	126370328	FAM53B	A/G	0.22 (0.06)	2.9E-04	0.23 (0.06)	4.0E-05	0.18 (0.14)	0.18	-0.23 (0.15)	0.12	0.19 (0.04)	4.6E-07
rs1006368	10	126336593	FAM53B	A/G	0.22 (0.06)	2.8E-04	0.23 (0.06)	4.2E-05	0.17 (0.13)	0.21	-0.21 (0.15)	0.15	0.19 (0.04)	5.0E-07
rs9938149	16	86889141	ZNF469	A/C	0.13 (0.04)	2.6E-03	0.21 (0.05)	5.3E-06	0.08 (0.07)	0.28	0.01 (0.08)	0.93	0.13 (0.03)	5.2E-07
rs10901827	10	126495959	FAM175B	T/G	-0.20 (0.06)	7.6E-04	-0.22 (0.06)	7.1E-05	-0.31 (0.15)	0.04	0.34 (0.16)	0.04	-0.19 (0.04)	7.7E-07
rs1034200	13	22126691	FTHL7	T/G	0.20 (0.04)	4.5E-06	0.07 (0.05)	1.5E-01	0.15 (0.08)	0.06	NA	NA	0.14 (0.03)	1.6E-06
rs4894535	3	173478299	FNDC3B	T/C	-0.19 (0.05)	4.3E-04	-0.16 (0.05)	1.1E-03	-0.13 (0.12)	0.31	-0.01 (0.13)	0.94	-0.16 (0.03)	2.2E-06
rs9965653	18	56553865	hCG_1659830	T/C	-0.12 (0.04)	2.4E-03	-0.09 (0.04)	3.8E-02	-0.28 (0.07)	2.2E-05	0.01 (0.07)	0.92	-0.12 (0.03)	2.4E-06
rs11750296	5	141916656	FGF1	A/G	-0.11 (0.05)	4.1E-02	-0.17 (0.06)	4.6E-03	-0.29 (0.11)	0.01	-0.29 (0.11)	0.01	-0.17 (0.04)	2.6E-06
rs6496932	15	83626571	LOC642935	A/C	-0.17 (0.05)	6.8E-04	-0.13 (0.05)	5.4E-03	-0.12 (0.10)	0.22	-0.12 (0.10)	0.25	-0.14 (0.03)	3.5E-06

rs2721043	13	39976668	LOC646982	T/G	-0.26 (0.07)	1.8E-04	-0.16 (0.08)	3.9E-02	-0.26 (0.17)	0.13	-0.24 (0.19)	0.22	-0.22 (0.05)	4.3E-06
rs2334946	13	22126060	FTHL7	T/C	-0.20 (0.04)	4.6E-06	-0.07 (0.05)	1.3E-01	-0.14 (0.08)	0.06	0.01 (0.08)	0.95	-0.13 (0.03)	5.3E-06
rs1548210	7	3493211	SDK1	T/C	-0.11 (0.04)	4.6E-03	-0.13 (0.04)	2.3E-04	-0.05 (0.07)	0.45	-0.03 (0.07)	0.71	-0.10 (0.02)	6.3E-06
rs33962	5	141909735	FGF1	A/G	0.08 (0.05)	8.9E-02	0.12 (0.05)	2.6E-02	0.28 (0.08)	7.8E-04	0.23 (0.09)	0.01	0.14 (0.03)	7.1E-06
rs11875795	18	42767069	KATNAL2	A/G	-0.25 (0.07)	5.2E-04	-0.12 (0.06)	4.8E-02	-0.55 (0.17)	1.5E-03	NA	NA	-0.20 (0.05)	8.2E-06
rs7204132	16	86902018	ZNF469	T/G	-0.09 (0.04)	2.5E-02	-0.18 (0.04)	5.4E-06	-0.05 (0.17)	0.55	0.03 (0.08)	0.76	-0.11 (0.03)	8.3E-06
rs7236403	18	42676659	PIAS2	T/C	0.25 (0.07)	6.4E-04	0.10 (0.07)	1.2E-01	0.45 (0.18)	0.01	0.43 (0.18)	0.02	0.20 (0.05)	8.6E-06
rs17138064	17	32263204	LOC727853	A/G	0.18 (0.07)	1.0E-02	0.28 (0.09)	1.0E-03	0.29 (0.17)	0.09	0.14 (0.19)	0.46	0.22 (0.05)	8.8E-06
rs7044529	9	136707872	COL5A1	T/C	-0.11 (0.06)	5.3E-02	-0.17 (0.05)	9.6E-04	-0.14 (0.12)	0.24	-0.30 (0.12)	0.02	-0.15 (0.03)	9.3E-06
